# *In Situ* Synthesis of Surface-Mounted Novel Nickel(II) Trimer-Based MOF on Nickel Oxide Hydroxide Heterostructures for Enhanced Methanol Electro-Oxidation

**DOI:** 10.3389/fchem.2021.780688

**Published:** 2021-11-29

**Authors:** Ya-Ya Sun, Yan-Jiang Wang, Qiu Pi, Ya-Pan Wu, Xue-Qian Wu, Shuang Li, Ya-Qian Lan, Qichun Zhang, Dong-Sheng Li

**Affiliations:** ^1^ College of Materials and Chemical Engineering, Key Laboratory of Inorganic Nonmetallic Crystalline and Energy Conversion Materials, China Three Gorges University, Yichang, China; ^2^ Hubei Three Gorges Laboratory, Yichang, China

**Keywords:** metal-organic framework, crystal structure, nickel oxide hydroxide, *in situ* synthesis, heterogeneous composites, methanol oxidation

## Abstract

Engineering the heterogeneous interface fusing MOFs and inorganic active component is an effective strategy to improve the electrochemical performance. Herein, we report a new Ni_3_-based MOF (denoted as CTGU-24) with an infrequent two-fold interpenetrating 3D (3,8)-connected network constructed from Ni(II) trimer and mixed tripodal tectonics for the electrocatalytic methanol oxidation reaction (MOR). In order to improve its stability and activities, the heterogeneous hybrid CTGU-24@NiOOH has been fabricated successfully *via* the first preparation of the NiOOH nanosphere and then *in situ* formation of CTGU-24 decorated on the NiOOH surface. Moreover, the integration of CTGU-24@NiOOH and different additives [acetylene black (AB) and ketjen black (KB)], resulting in the optimized hybrid sample AB&CTGU-24@NiOOH (4:4). It attains better MOR performance with an area-specific peak current density of 34.53 mA·cm^−2^ than pure CTGU-24 (14.99 mA·cm^−2^) and improved durability in an alkali medium. The new findings indicate that the CTGU-24@NiOOH heterostructure formed *in situ* and the integration of moderate additives are critical to optimizing and improving electrocatalytic activity of pure MOF crystalline material.

## Introduction

The development of new, economical, and recyclable energy materials to reduce the consumption of fossil fuels and their harmful effects on the natural environment and human beings has become a research focus in recent decades. Direct methanol fuel cells (DMFCs), with their advantages of high efficiency, high energy density, and low cost, have attracted extensive attention in the field of the sustainable energy conversion scheme (Wu X. et al., 2019; Dao et al., 2019; Li H. et al., 2020; Zhu et al., 2020). The methanol oxidation reaction (MOR) requires efficient electrocatalysts to promote sluggish reaction kinetics (Wang Q. et al., 2020). So far, noble metals and their alloy Ru/Ir-based complexes are the most widely used and efficient electrocatalysts for the MOR (Zhu et al., 2015; Kwon et al., 2018; Gong et al., 2019; Li M. et al., 2020). However, the high cost, scarce supply, and being easily poisoned by CO largely restrict their practical applications in the MOR process (Cui et al., 2017; Li et al., 2018; Dubale et al., 2020; Yuda et al., 2020). Therefore, the search for efficient, durable, and most importantly, inexpensive alternative materials to noble metal-based catalysts is of utmost importance.

Among the large number of catalyst materials, nickel-based materials, such as nanostructured nickel oxide, nickelous hydroxide, nickel oxyhydroxide, and their heterostructures with available oxidation reduction state of Ni^2+^ and Ni^3+^, have been confirmed to display promising efficient MOR activity under alkaline environments (Li et al., 2017; Bott-Neto et al., 2019; Wang T.-J. et al., 2019; Liu et al., 2021; Wang et al., 2021). Nevertheless, the substantial changes in MOR performance are needed to match the kinetic superiority of platinum-based materials (Mansor et al., 2019). Metal-organic frameworks (MOFs) have been extensively studied since it was invented because of its adjustable structure, specific surface area, and diversified functions (Kirchon et al., 2018). Considering that the diverse metal nodes of MOFs can provide high-density catalytic sites, the reasonable design of active units might provide a common approach for efficient applications including oxygen evolution reaction, urea oxidation, and MOR processes (Zhu et al., 2017; Aiyappa et al., 2019; Wu Y. P. et al., 2019; Wang X. et al., 2020). However, the inadequate chemical stability and poor electrical conductivity greatly restrict its electrocatalytic performance. In this context, one of the effective strategies to enhance the MOR activities is by generating MOF heterostructures with inorganic nanomaterials and further integrating the conductive additives, which are favorable for the electron transfer and reactivity. Our recent studies confirmed that the design and synthesis of a new type of Ni-based MOFs and their hybrid structures show impressive electron transfer efficiency and reaction activity, resulting in improved electrochemical performance. However, there are still great challenges in designing and synthesizing MOF materials with novel and well-defined structures and preparing heterogeneous composites. Herein, a novel Ni_3_-based MOF [Ni_3_ (μ_3_-OH) (Me_2_NH_2_) (BTB)_2_(TPT)] (CTGU-24) has been prepared successfully based on 1,3,5-tri(4-carboxyphenyl)benzene (H_3_BTB), 2,4,6-triste (4-pyridyl)-1,3,5-triazine(TPT), and [Ni_3_(μ_3_-OH) (COO)_6_] clusters. Besides, the CTGU-24@NiOOH heterostructure has conveniently designed an *in situ* formation of CTGU-24 decorated on the NiOOH surface. The CTGU-24@NiOOH heterogeneous sample possesses improved MOR activity with the area-specific peak current density of 18.75 mA cm^−2^ compared with CTGU-24 (14.99 mA·cm^−2^). Subsequently, a series of hybrid materials were prepared through the integration of different conductive additives such as AB and KB with the above composite. Strikingly, the as-synthesized AB&CTGU-24@NiOOH (4:4) shows remarkable MOR activities with a high peak current density of 34.53 mA·cm^−2^. This work can provide new perspective to design efficient MOF-based heterostructure materials for the applications in energy storage and electrocatalysis.

## Materials and methods

### Materials

All reagents are of analytical purity and used without further purification. Nickel perchlorate hexahydrate (Ni(ClO_4_)_2_·6H_2_O), nickelous hydroxide (Ni(OH)_2_), potassium peroxydisulfate (K_2_S_2_ O_8_), sodium hydroxide (NaOH), 3,5-tri(4-carboxyphenyl)benzene (H_3_BTB), and 2,4,6-triste (4-pyridyl)-1,3,5-triazine (TPT) were purchased from Alfa Aesar (Haverhill, MA, USA). Other chemicals such as dimethylacetamide (DMA), fluoboric acid (HBF_4_), acetylene black (AB), and ketjen black (KB) were purchased from Sinopharm Chemical Reagent Co., Ltd (Shanghai, China). All aqueous solutions were performed using deionized water.

### Synthetic procedures

#### Preparation of [Ni_3_(μ_3_-OH) (Me_2_NH_2_) (BTB)_2_(TPT)] (CTGU-24)

Solvothermal reactions of Ni (ClO_4_)_2_.6H_2_O (36.5 mg, 0.1 mmol), H_3_BTB (11.0 mg, 0.025 mmol), and TPT (3.0 mg, 0.01 mmol) in 5.5 ml of DMA/H_2_O (v/v = 5/0.5) and three drops of HBF_4_ were added in a 25-ml Teflon-lined stainless steel vessel. The mixture was kept at 120°C for 48 h and then cooled to room temperature. Green block crystals were collected and washed with DMA and water. (Yield: 48%, based on nickel.) Elemental analysis (calcd) found for C_74_H_51_N_7_Ni_3_O_13_: C 62.49, H 3.61, N 6.89; Found: C 62.85, H3.74, N 6.93.

#### Preparation of the NiOOH precursor

Typically, 1.4 g of Ni(OH)_2_ was added into 100 ml 1.0 M NaOH aqueous solution under stirring, then 6.0 g K_2_S_2_O_8_ was added to the mixed solution and reacted at room temperature for 24 h. The product was washed three times with deionized water and ethanol respectively and dried at 80°C under vacuum overnight.

#### Preparation of CTGU-24@NiOOH composites

Similar to the synthesis of CTGU-24, in a 25-ml Teflon-lined stainless-steel vessel, 30 mg of H_3_BTB, 10 mg of TPT, and 50 mg of NiOOH precursor were dispersed in 10 ml of DMA, 1.0 ml of water, and six drops of HBF_4_. The vial was tightly closed and put into an oven at 120°C for 48 h. After being cooled to room temperature, the products were washed three times with DMA and water. The samples were heated under vacuum to 80°C for 12 h.

#### Preparation of AB&CTGU-24@NiOOH hybrid catalysts

The different masses of acetylene black (AB) additive such as 10, 20, 30, and 40 mg were mixed with 40 mg of as-synthesized CTGU-24@NiOOH and mechanically ground for 30 min, the as-prepared hybrid materials are denoted as AB&CTGU-24@NiOOH(1:4), AB&CTGU-24@NiOOH(2:4), AB&CTGU-24@NiOOH(3:4), and AB&CTGU-24@NiOOH(4:4), respectively. For comparison, another series of KB&CTGU-24@NiOOH hybrid catalysts were prepared by a similar method. Finally, the composites were confirmed by PXRD measurements.

### Methods

#### Characterization

Powder X-ray diffraction (PXRD) patterns was studied on a Rigaku Ultima IV diffractometer (Cu Kα radiation, λ = 1.5406 Å, 2θ range of 5°–80°, and a scan rate of 8 min^−1^). Thermogravimetric (TG) curves were performed on a Netzsch model 449°C thermal analyzer heated from 25°C to 800°C with a heating rate of 10°C min^−1^ under an air atmosphere. The morphologies and microstructures were observed by field-emission scanning electron microscopy (FE-SEM; JEOL JSM-7500F, Tokyo, Japan) operating at 15 kV. The morphologies and microstructures were assessed using transmission electron microscopy (TEM; JEOL F200, Japan) with an accelerating voltage of 200 kV. X-ray photoelectron spectroscopy (XPS) measurements were conducted to study the chemical state of the elements of pure CTGU-24 and CTGU-24@NiOOH using a monochromatized Al Kα X-ray as the excitation source (ESCALAB MKII), and the binding energies (BEs) were calibrated by C 1 s to 284.8 eV.

#### X-ray crystallography

Single-crystal diffraction data of CTGU-24 were collected on a Rigaku XtaLAB diffractometer with Cu *Kα* radiation (λ = 1.5418 Å) at 293 K. The structure was solved by a direct method and refined by full-matrix least squares using SHELX-2014 and OLEX2.0-1.2. The structure was solved with the ShelXT structure solution program and refined with the ShelXL refinement package using least square minimization. All non-hydrogen atoms were refined optimally with anisotropy. All hydrogen atoms were in ideal positions after being refined. Crystallographic data (excluding structure factors) for CTGU-24 in this paper have been deposited with the Cambridge Crystallographic Data Centre (CCDC, 12 Union Road, Cambridge CB21EZ, UK). Copies of the data can be obtained free of charge on quoting the depository numbers CCDC-2109470 for CTGU-24 (http://www.ccdc.cam.ac.uk). Crystal data and structure refinements and selected bond distances and angles for CTGU-24 are summarized in Supplementary Tables S1, S2.

#### Electrochemical measurements

The electrochemical measurements were carried out on a CHI660e electrochemical workstation at room temperature. All measurements were carried out in a three-electrode electrochemical cell. The working electrode was a glassy carbon electrode (3 mm inner diameter, 0.0706 cm^−2^), the Hg/HgO electrode was used as the reference electrode, and a Pt wire was used as the counter electrode, respectively. Generally, 4 mg of catalyst was dispersed in 0.5 ml ethanol and 1.5 ml deionized water containing 0.2 ml 5wt% Nafion and sonicated for 30 min. Then, 4 μl of the catalyst ink was loaded onto the GCE and then dried at room temperature. The cyclic voltammetry (CV) measurement was typically carried out in N_2_-saturated 0.1 M KOH solution, and the activity of MOR was recorded in N_2_-saturated 0.1 M KOH containing 1.0 M CH_3_OH electrolyte with a scan rate of 50 mV s^−1^. All the electrochemical data were used without *iR* correction. The mass activity of catalysts, defined as the peak current per amount of metal loading, was evaluated the catalytic performances of different samples.

## Results and discussion

### Description of structure of CTGU-24

**FIGURE 1:**
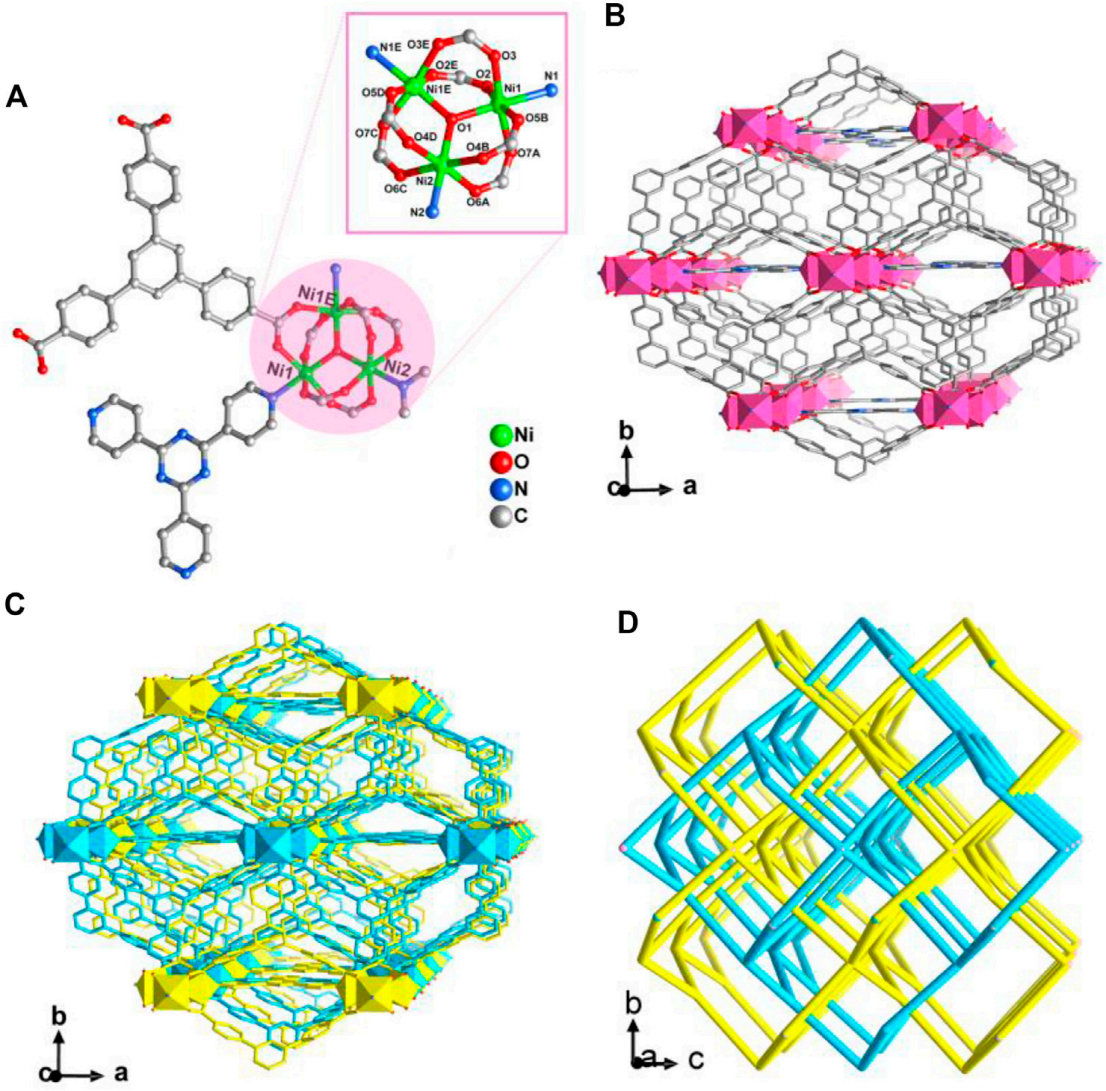
**(A)** View of the [Ni_3_(μ_3_-OH) (COO)_6_] cluster unit in CTGU-24. The inset is the secondary building unit (SBU) of CTGU-24. **(B)** View of the single 3D framework of CTGU-24 along the *c* axis. **(C)** View of the two-fold interpenetrating 3D frameworks of CTGU-24 along the *c* axis. **(D)** Schematic representation of the 3D two-fold interpenetrating (3,8)-connected network of CTGU-24 (the single network is shown by cyanine and yellow lines).

CTGU-24 crystallizes in the orthorhombic space group *C*mc2_1_ and presents an infrequent two-fold interpenetrating 3D (3,8)-connected network constructed from Ni(II) trimer and mixed tripodal tectonics. The asymmetric unit contains two crystallographic independent Ni^II^ ions, one BTB ligand, a half TPT molecule, a half Me_2_NH_2_^+^ ion generated by *in-situ* decomposition of DMA solvent, and a half µ_3_-OH ion (Figure 1A). In CTGU-24, Ni1, Ni2, and N1E linked six carboxylate groups and a hydroxyl bridge to generate a typical [Ni_3_(μ_3_-OH) (COO)_6_] cluster unit. Finally, the adjacent [Ni_3_(μ_3_-OH) (COO)_6_] clusters are bridged by six BTB ligands and two TPT molecules to form a 3D open framework along the c axis (Figure 1B). Besides, owing to the presence of enough space, two such frameworks are interweaved into a two-fold interpenetrating array (Figure 1C). Topologically, by regarding each [Ni_3_(μ_3_-OH) (COO)_6_] cluster as an eight-connected node, the BTB ligand are simplified as 3-c nodes, and the overall 3D framework can be rationalized as two-fold interpenetrating (3,8)-connected nets with a point symbol of (3·5^2^)_2_(3^4^·4^2^·5^6^·6^12^·7^3^·8) (Figure 1D).

### Thermal stabilities, PXRD, and FT-IR analysis

**FIGURE 2:**
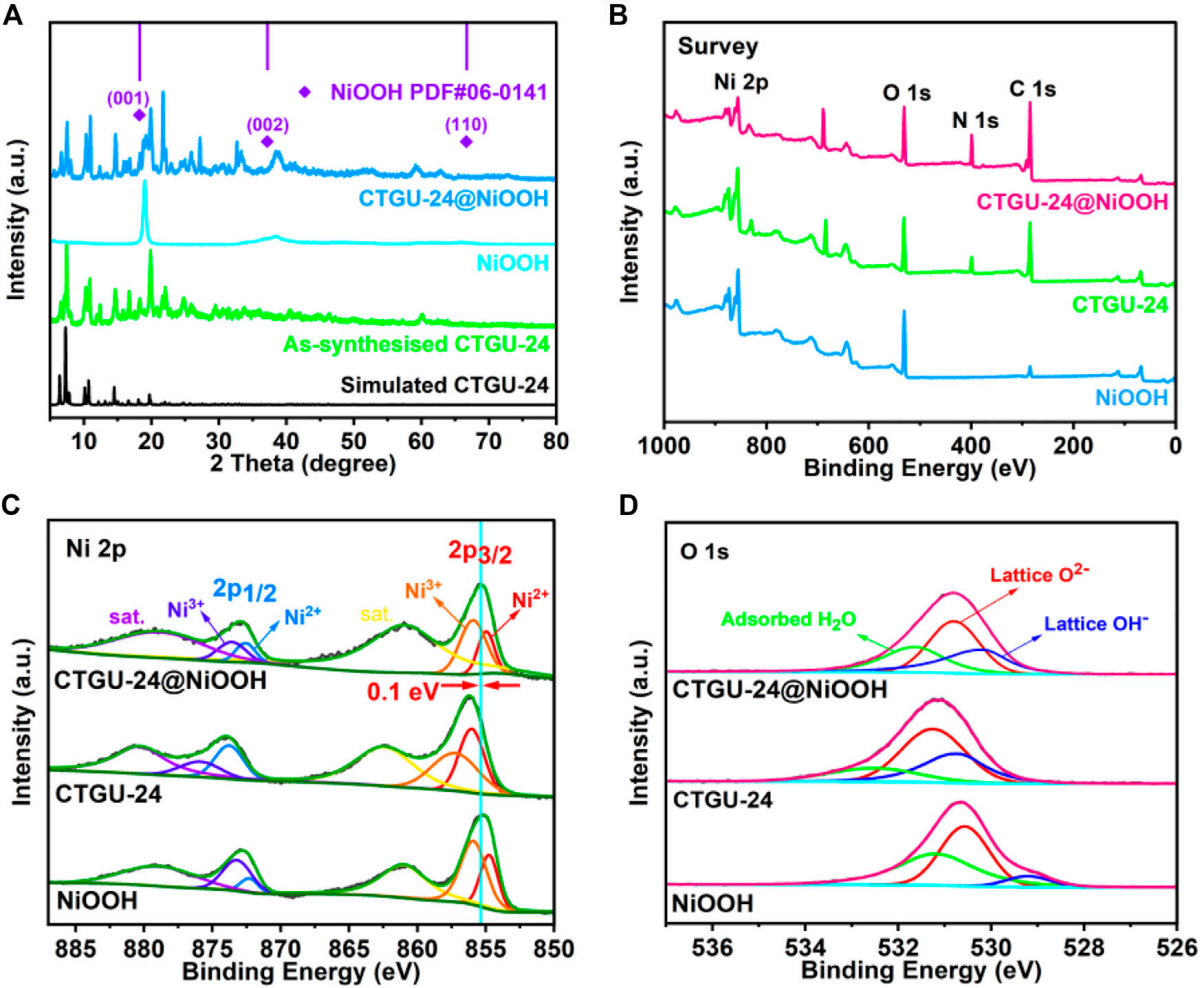
**(A)** PXRD patterns of simulated CTGU-24, as-synthesized CTGU-24, NiOOH, and CTGU-24@NiOOH. XPS of the survey scan **(B**, **C)** Ni 2p and **(D)** O 1s of NiOOH, CTGU-24, and CTGU-24@NiOOH.

Thermogravimetric analysis (TGA) was studied for CTGU-24 to investigate the thermal stability (Supplementary Figure S1). CTGU-24 exhibits a plateau of stability up to about 160°C, and then the framework begins to collapse. The complete collapse of CTGU-24 can be observed at around 400°C, which is accompanied by decomposition of the whole CTGU-24 skeleton. To confirm the phase purity of the bulk materials, the PXRD patterns show that the diffraction peaks of both the simulated and experimental patterns match well in relevant positions, indicating that CTGU-24 has good crystallinity and higher phase purity (Figure 2A). Besides, the heterostructures CTGU-24@NiOOH were prepared by a one-pot well-controlled solvothermal reaction of the obtained NiOOH precursor with BTB and TPT ligands. PXRD was used to confirm the successful phase transformation of the as-obtained samples. As displayed in Figure 2A, the main diffraction peaks of the NiOOH precursor can be indexed to pure NiOOH phase (PDF No. 06-0141). With the introduction of BTB and TPT, some characteristic peaks (sharp peak location: 6.4°, 7.3°, 10.1°, 10.7°, 14.5°, and 19.7°) of CTGU-24 can be identified, which indicates the successful formation of CTGU-24 constructed from mixed ligands and NiOOH. Furthermore, the patterns of AB&CTGU-24@NiOOH (4:4), KB&CTGU-24@NiOOH (4:4), and CTGU-24@NiOOH samples reveal dispersing diffraction peaks at around 25°, which is attributed to the introduction of two conductive additives (Supplementary Figure S2). Moreover, the FT-IR spectrum of CTGU-24@NiOOH composites shows the similar characteristic absorption bands with CTGU-24; in particular, CTGU-24@NiOOH still retains the characteristic absorption peak of OH^−^ at 3,600 cm^−1^, which corresponds to NiOOH (Supplementary Figure S3).

### Morphologies and XPS characterization

**FIGURE 3:**
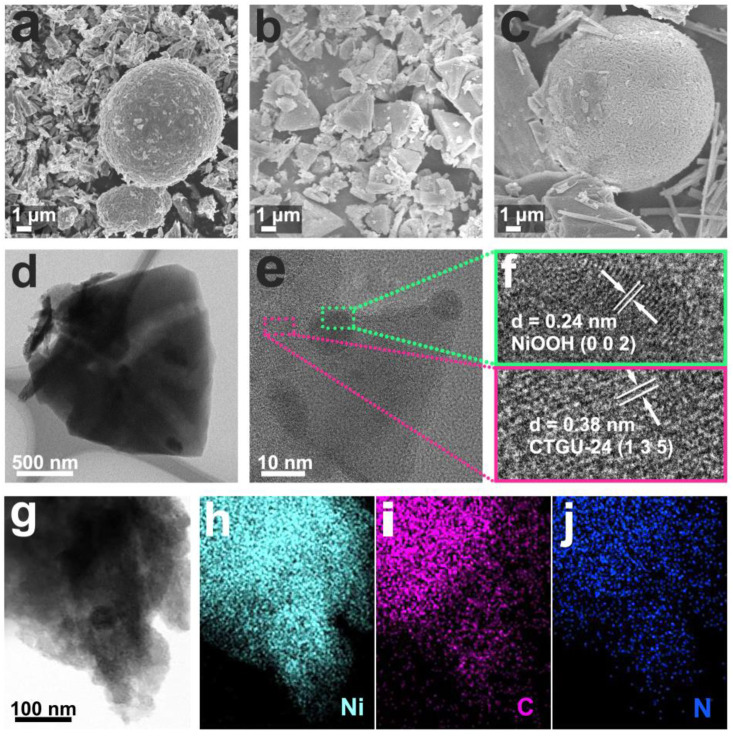
Morphology and structure characterizations of NiOOH, CTGU-24 and CTGU-24@NiOOH. **(A)** SEM image of NiOOH. **(B)** SEM image of CTGU-24. **(C)** SEM image of CTGU-24@NiOOH. **(D, G)** HRTEM image of CTGU-24@NiOOH, and STEM-EDX elemental mapping of CTGU-24@NiOOH for **(H)** Ni, **(I)** C, and **(J)** *N* elements.

The physical and morphological characterizations of NiOOH, CTGU-24, and CTGU-24@NiOOH are shown in Figure 3. As a contrast, the structural features of pure CTGU-24 were characterized by using SEM and TEM. Supplementary Figure S4A confirms the triangle morphology of CTGU-24. TEM images reveal that CTGU-24 retained the good crystalline morphology with SEM (Supplementary Figures S4B–D). Figures 3A,B show the SEM images of the pure NiOOH and CTGU-24; compared with pure NiOOH, the CTGU-24 particles are uniformly and regularly grown on the surface of NiOOH nanospheres, thereby forming a CTGU-24@NiOOH heterostructure after solvothermal reaction. The transformation of the CTGU-24 morphology may be caused by the combined effect of the NiOOH precursor and the increase of organic ligand on the reaction conditions. Transmission electron microscopy (TEM) was used to further assess the morphology of CTGU-24@NiOOH material. As shown in Figures 3E,F, the high-resolution TEM (HRTEM) clearly shows a hetero-interface with the lattice fringes of 0.24 and 0.38 nm, which might be ascribed to the (135) plane of CTGU-24 (pink box) and the (002) plane of NiOOH (green box), respectively, indicating the formation of the CTGU-24@NiOOH heterojunction. Furthermore, high-angle annular dark-field TEM (HAADF-STEM) and elemental mapping images reveal that Ni and N elements are well distributed throughout the corresponding range of the composites in Figures 3H–J.

XPS was used to investigate the surface composition and valence states of the as-prepared samples containing Ni, O, N, and C as in the survey spectra shown in Figure 2 and Supplementary Figure S5. Analysis of the Ni 2p spectrum (Figure 2C) suggests that there are two chemical states in NiOOH and CTGU-24@NiOOH, corresponding to Ni^2+^ at 855.02(2p_3/2_) and 872.51(2p_1/2_) eV and Ni^3+^ at 855.96(2p_3/2_) and 873.73(2p_1/2_) eV. For CTGU-24, only the Ni^2+^ signals are observed, suggesting that the sample of CTGU-24@NiOOH contains both CTGU-24 and NiOOH (Wang B. et al., 2019; Li et al., 2019). Besides, in Figure 2D, the O 1 s spectrum for the composite of CTGU-24@NiOOH combines the characteristics of NiOOH and CTGU-24, which may be due to the interaction between the two phases. Compared with NiOOH, the displacements of the two kinds of Ni on 2P_3/2_ are +0.23 eV and +0.16 eV, respectively, while the displacements on 2p_1/2_ are +0.1 eV and +0.35 eV. Due to these shifts, the total shift of the binding energy of CTGU-24@NiOOH on the Ni 2p peak is 0.1 eV in contrast to the NiOOH precursor, which is also proved that CTGU-24@NiOOH inherits the original Ni ionic valence state of NiOOH. The interaction between organic ligands and Ni ions released from NiOOH results in the adjustment of the local electron cloud structure, which may enhance the catalytic activity for MOR. Consequently, better electrochemical MOR performances can be expected for CTGU-24@NiOOH.

### Evaluation of MOR activities

**FIGURE 4:**
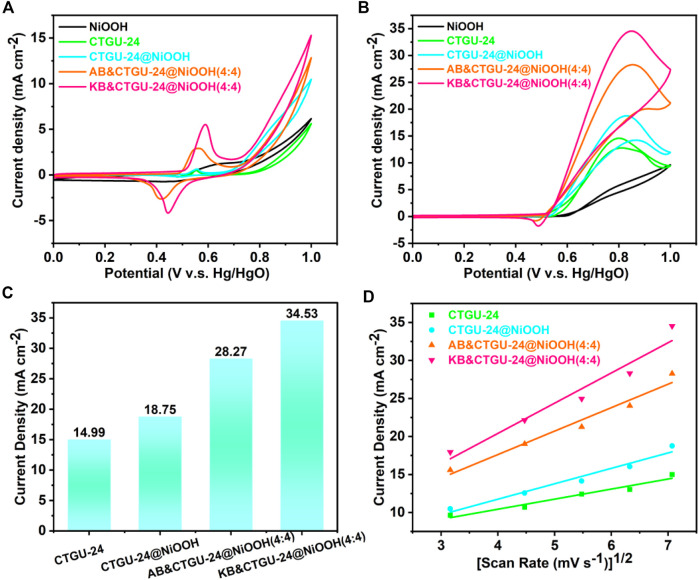
CV curves of four as-synthesized catalysts in 0.1 M KOH in the absence **(A)** and **(B)** presence of 1.0 M methanol at a scan rate of 50 mV·s^−1^. **(C)** Bar graph of the area current density for four samples. **(D)** View of the linear relationship between the current density and the square root of the scan rate for four catalysts.

To demonstrate the structural benefits of the CTGU-24@NiOOH hybrid material, MOR was carried out to evaluate the performance of NiOOH, CTGU-24at NiOOH, and CTGU-24@NiOOH composites. The series of composites such as AB&CTGU-24@NiOOH and KB&CTGU-24@NiOOH were also assessed for comparison. Typically, the electrocatalytic MOR measurements of as-prepared samples were performed in 0.1 M KOH with and without 1.0 M methanol solution. All the catalysts were loaded on GCE which acts as the working electrode, and their current densities were normalized by the area activity. Figure 4A shows the CV curves of pure CTGU-24, CTGU-24@NiOOH, AB& CTGU-24@NiOOH (4:4), and KB&CTGU-24@NiOOH (4:4) in 0.1 M KOH solution at a scan rate of 50 mV s^−1^. It could be observed that the obvious redox peaks correspond to Ni^2+^/Ni^3+^ in the range of 0.552–0.589 V in the four samples. The electrocatalytic activity of four catalysts toward MOR is investigated in the solution of 1.0 M KOH+1.0 M CH_3_OH (Figure 4B). All as-prepared samples exhibit distinct catalytic activity for methanol oxidation. Moreover, KB&CTGU-24@NiOOH (4:4) has the highest area activity (34.53 mA cm^−2^), which is larger than those of KB&CTGU-24@NiOOH (4:4) (28.27 mA cm^−2^) and CTGU-24@NiOOH (18.75 mA cm^−2^) and around 2.3 times higher than that of pure CTGU-24 (14.99 mA cm^−2^) (Figure 4C), indicating its superior catalytic activity for MOR, which is mainly attributed to the synergistic effect between heterogeneous CTGU-24@NiOOH and conductive additives.

Additionally, to clarify the factors contributing to the MOR activities of CTGU-24@NiOOH, the MOR properties of series of hybrid materials made from CTGU-24@NiOOH, AB, and KB conducting materials were also studied (Supplementary Table S4). The CV curves for activating catalysts in 0.1 M KOH were obtained for a series of AB& and KB&CTGU-24@NiOOH composites (1:4, 2:4, 3:4, and 4:4) (Supplementary Figures S6, S9). Furthermore, the peaks for as-synthesized composites at around 0.83 V obviously belong to the MOR, when measured in 0.1 M KOH +1.0 M CH_3_OH solution (Supplementary Figures S6B, S9B). Noteworthy is that the AB&CTGU-24@NiOOH (2:4) and KB&CTGU-24@NiOOH (4:4) materials show the highest current density of 29.87 and 34.53 mA cm^−2^ in the forward scan, which is larger than those of other composites. This improved MOR activity might result from the enhanced electric conductivity in the presence of AB and KB. In the meantime, the peak current densities of all as-prepared samples increase linearly with the square root of the scan rate for sweep rates from 10 to 50 mV s^−1^ (Figure 4D, Supplementary Figures S6D, S9D), which indicates that their MOR process was determined by the same diffusion speed. To emphasize the outstanding performance of CTGU-24@NiOOH, a comparison with recently reported active MOR catalysts is presented in Supplementary Table S4. These findings might elucidate that CTGU-24@NiOOH heterostructures constructed *via* a self-sacrificing template process not only inherit the large surface area of MOF but also have excellent phase interfaces with electrolyte. Moreover, the integration of carbon materials such as AB and KB improves the electrical conductivity of CTGU-24@NiOOH composite and accelerates the electrooxidation of methanol.

**FIGURE 5:**
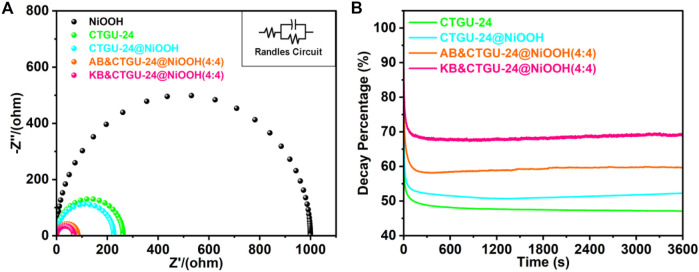
**(A)** Nyquist plots of four as-synthesized catalysts. The insets is the equivalent circuit. **(B)** chronoamperograms for four catalysts.

Besides, the electrode kinetics of as-prepared samples in the MOR process was investigated by electrochemical impedance spectroscopy (EIS) (Figure 5, Supplementary Figures S8, S11). The EIS Nyquist curves for four catalysts were carried out at onset potentials (Figure 5A). Also, the Rct value of KB&CTGU-24@NiOOH (4:4) (68.64 Ω) was smaller than those of the other three samples, disclosing that KB& CTGU-24@NiOOH (4:4) could be beneficial to the fast electron transport efficiency and improved MOR kinetics. Finally, the chronoamperometry test was used to study the durability of four samples in 0.1 M KOH in the presence of 1.0 M CH_3_OH. As shown in Figure 5B, the current densities dropped at the very beginning and then almost kept no changes over 3,600 s for the four samples. The same phenomenon was always observed in reported studies, which could result from the poisoning of catalysts caused by the intermediate species. Apparently, KB&CTGU-24@NiOOH (4:4) shows the smallest degree of reduction compared with the other three catalysts, which could benefit from the coupling effect between CTGU-24@NiOOH and moderate addition of KB with the excellent MOR activities. Additionally, some characterizations including SEM (Supplementary Figure S12), XPS (Supplementary Figure S13), and XRD (Supplementary Figures S14, S15) also confirmed the persistence of CTGU-24@NiOOH and KB&CTGU-24@NiOOH (4:4) after MOR tests. Generally, the above results demonstrate that KB&CTGU-24@NiOOH (4:4) attains outstanding MOR performance and good durability.

## Conclusion

In summary, a new nickel(II) trimer-based MOF has been synthesized and structurally characterized. Besides, the hierarchical CTGU-24@NiOOH structure and its composite *via* a simple semi-transformation from NiOOH as efficient electrode materials for MOR were analyzed. The optimized hybrid material KB&CTGU-24@NiOOH (4:4) shows the highest electrocatalytic MOR activity compared to the pure CTGU-24 electrocatalyst. The distinctive characteristics include diversity of components and synergistic complementarity, endowing KB& CTGU-24@NiOOH with superior MOR activity and endurance. This work paves a new way for designing highly efficient Ni-based heterogeneous electrocatalysts for methanol oxidation and could attract more scientific interest in new cluster-based MOF materials for the field of energy conversion.

## Data Availability

The original contributions presented in the study are included in the article/Supplementary Material; further inquiries can be directed to the corresponding authors.
