# Supplementary material for: In Situ Synthesis of Surface-Mounted Novel Nickel(II) Trimer-Based MOF on Nickel Oxide Hydroxide Heterostructures for Enhanced Methanol Electro-Oxidation
Source: Front Chem. 2021 Nov 29;9:780688. doi: 10.3389/fchem.2021.780688 (PMC8666528; doi:10.3389/fchem.2021.780688)

checkCIF/PLATON report

Structure factors have been supplied for datablock(s) ctgu-24

THIS REPORT IS FOR GUIDANCE ONLY. IF USED AS PART OF A REVIEW PROCEDURE FOR PUBLICATION, IT SHOULD NOT REPLACE THE EXPERTISE OF AN EXPERIENCED CRYSTALLOGRAPHIC REFEREE.

No syntax errors found. CIF dictionary Interpreting this report

Datablock: ctgu-24

```
Bond precision:   C-C = 0.0047 A          Wavelength=1.54184

Cell:              a=16.7054(3)      b=24.2139(4)      c=22.5829(4)
                   alpha=90          beta=90           gamma=90
Temperature:       290 K
```

	Calculated	Reported
Volume	9134.9(3)	9134.8(3)
Space group	C m c 21	C m c 21
Hall group	C 2c -2	C 2c -2
Moiety formula	C74 H49 N7 Ni3 O13 [+ solvent]	C74 H49 N7 Ni3 O13
Sum formula	C74 H49 N7 Ni3 O13 [+ solvent]	C74 H49 N7 Ni3 O13
Mr	1420.27	1420.33
Dx,g cm-3	1.033	1.033
Z	4	4
Mu (mm-1)	1.144	1.144
F000	2920.0	2920.0
F000’	2892.19	
h,k,lmax	20,30,28	20,30,27
Nref	9673[4967]	6926
Tmin,Tmax	0.789,0.786	0.611,1.000
Tmin’	0.715	

```
Correction method= # Reported T Limits: Tmin=0.611 Tmax=1.000
AbsCorr = MULTI-SCAN

Data completeness= 1.39/0.72        Theta(max)= 74.362
```

R(reflections)= 0.0298(6382)   wR2(reflections)= 0.0823(6926)

S = 1.036   Npar= 464

The following ALERTS were generated. Each ALERT has the format
test-name_ALERT_alert-type_alert-level.
Click on the hyperlinks for more details of the test.

Alert level C

```
PLAT213_ALERT_2_C Atom N2              has ADP max/min Ratio .....        3.9 prolat
PLAT213_ALERT_2_C Atom C1              has ADP max/min Ratio .....        3.2 prolat
PLAT213_ALERT_2_C Atom C21             has ADP max/min Ratio .....        3.2 prolat
PLAT220_ALERT_2_C NonSolvent   Resd 1  C   Ueq(max)/Ueq(min) Range        4.1 Ratio
PLAT220_ALERT_2_C NonSolvent   Resd 1  N   Ueq(max)/Ueq(min) Range        3.2 Ratio
PLAT222_ALERT_3_C NonSolvent Resd 1   H   Uiso(max)/Uiso(min) Range       5.0 Ratio
PLAT241_ALERT_2_C High   'MainMol' Ueq as Compared to Neighbors of         O2 Check
PLAT241_ALERT_2_C High   'MainMol' Ueq as Compared to Neighbors of         O3 Check
PLAT241_ALERT_2_C High   'MainMol' Ueq as Compared to Neighbors of         N2 Check
PLAT241_ALERT_2_C High   'MainMol' Ueq as Compared to Neighbors of         C4 Check
PLAT241_ALERT_2_C High   'MainMol' Ueq as Compared to Neighbors of         C5 Check
PLAT241_ALERT_2_C High   'MainMol' Ueq as Compared to Neighbors of        C20 Check
PLAT241_ALERT_2_C High   'MainMol' Ueq as Compared to Neighbors of        C21 Check
PLAT241_ALERT_2_C High   'MainMol' Ueq as Compared to Neighbors of        C29 Check
PLAT241_ALERT_2_C High   'MainMol' Ueq as Compared to Neighbors of        C30 Check
PLAT242_ALERT_2_C Low    'MainMol' Ueq as Compared to Neighbors of        Ni1 Check
PLAT242_ALERT_2_C Low    'MainMol' Ueq as Compared to Neighbors of        Ni2 Check
PLAT242_ALERT_2_C Low    'MainMol' Ueq as Compared to Neighbors of         C2 Check
PLAT242_ALERT_2_C Low    'MainMol' Ueq as Compared to Neighbors of         C3 Check
PLAT242_ALERT_2_C Low    'MainMol' Ueq as Compared to Neighbors of         C6 Check
PLAT242_ALERT_2_C Low    'MainMol' Ueq as Compared to Neighbors of        C17 Check
PLAT242_ALERT_2_C Low    'MainMol' Ueq as Compared to Neighbors of        C19 Check
PLAT242_ALERT_2_C Low    'MainMol' Ueq as Compared to Neighbors of        C22 Check
PLAT242_ALERT_2_C Low    'MainMol' Ueq as Compared to Neighbors of        C33 Check
PLAT250_ALERT_2_C Large U3/U1 Ratio for Average U(i,j) Tensor ....        2.5 Note
PLAT334_ALERT_2_C Small Aver. Benzene C-C Dist C27      -C29             1.37 Ang.
PLAT410_ALERT_2_C Short Intra H...H Contact  H5       ..H8       .       1.99 Ang.
                                                    x,y,z  =      1_555 Check
PLAT430_ALERT_2_C Short Inter D...A Contact  O5       ..O6       .       2.86 Ang.
                                                1-x,1+y,z  =      4_665 Check
PLAT911_ALERT_3_C Missing FCF Refl Between Thmin & STh/L=    0.600         21 Report
```

Alert level G

```
PLAT004_ALERT_5_G Polymeric Structure Found with Maximum Dimension          3 Info
PLAT007_ALERT_5_G Number of Unrefined Donor-H Atoms .............           1 Report
PLAT303_ALERT_2_G Full Occupancy Atom H1         with # Connections      3.00 Check
PLAT606_ALERT_4_G Solvent Accessible VOID(S) in Structure ........          ! Info
PLAT794_ALERT_5_G Tentative Bond Valency for Ni1       (II)      .       2.16 Info
PLAT794_ALERT_5_G Tentative Bond Valency for Ni2       (II)      .       1.97 Info
PLAT910_ALERT_3_G Missing # of FCF Reflection(s) Below Theta(Min).          1 Note
PLAT912_ALERT_4_G Missing # of FCF Reflections Above STh/L=  0.600        130 Note
PLAT915_ALERT_3_G No Flack x Check Done: Low Friedel Pair Coverage         45 %
PLAT941_ALERT_3_G Average HKL Measurement Multiplicity ..........         3.2 Low
PLAT978_ALERT_2_G Number C-C Bonds with Positive Residual Density.          0 Info
```

```
   0 ALERT level A = Most likely a serious problem - resolve or explain
   0 ALERT level B = A potentially serious problem, consider carefully
  29 ALERT level C = Check. Ensure it is not caused by an omission or oversight
  11 ALERT level G = General information/check it is not something unexpected

   0 ALERT type 1 CIF construction/syntax error, inconsistent or missing data
  29 ALERT type 2 Indicator that the structure model may be wrong or deficient
   5 ALERT type 3 Indicator that the structure quality may be low
   2 ALERT type 4 Improvement, methodology, query or suggestion
   4 ALERT type 5 Informative message, check
```

Validation response form

Please find below a validation response form (VRF) that can be filled in and pasted into your CIF.

```
# start Validation Reply Form
_vrf_PLAT213_ctgu-24
;
PROBLEM: Atom N2              has ADP max/min Ratio .....        3.9 prolat
RESPONSE: ...
;
_vrf_PLAT220_ctgu-24
;
PROBLEM: NonSolvent   Resd 1  C   Ueq(max)/Ueq(min) Range        4.1 Ratio
RESPONSE: ...
;
_vrf_PLAT222_ctgu-24
;
PROBLEM: NonSolvent Resd 1  H   Uiso(max)/Uiso(min) Range        5.0 Ratio
RESPONSE: ...
;
_vrf_PLAT241_ctgu-24
;
PROBLEM: High   'MainMol' Ueq as Compared to Neighbors of         O2 Check
RESPONSE: ...
;
_vrf_PLAT242_ctgu-24
;
PROBLEM: Low    'MainMol' Ueq as Compared to Neighbors of        Ni1 Check
RESPONSE: ...
;
_vrf_PLAT250_ctgu-24
;
PROBLEM: Large U3/U1 Ratio for Average U(i,j) Tensor ....        2.5 Note
RESPONSE: ...
;
_vrf_PLAT334_ctgu-24
;
PROBLEM: Small Aver. Benzene C-C Dist C27      -C29             1.37 Ang.
RESPONSE: ...
;
_vrf_PLAT410_ctgu-24
;
PROBLEM: Short Intra H...H Contact  H5       ..H8       .       1.99 Ang.
RESPONSE: ...
```

```
;
_vrf_PLAT430_ctgu-24
;
PROBLEM: Short Inter D...A Contact  O5       ..O6       .       2.86 Ang.
RESPONSE: ...
;
_vrf_PLAT911_ctgu-24
;
PROBLEM: Missing FCF Refl Between Thmin & STh/L=    0.600         21 Report
RESPONSE: ...
;
# end Validation Reply Form
```

It is advisable to attempt to resolve as many as possible of the alerts in all categories. Often the minor alerts point to easily fixed oversights, errors and omissions in your CIF or refinement strategy, so attention to these fine details can be worthwhile. In order to resolve some of the more serious problems it may be necessary to carry out additional measurements or structure refinements. However, the purpose of your study may justify the reported deviations and the more serious of these should normally be commented upon in the discussion or experimental section of a paper or in the "special_details" fields of the CIF. checkCIF was carefully designed to identify outliers and unusual parameters, but every test has its limitations and alerts that are not important in a particular case may appear. Conversely, the absence of alerts does not guarantee there are no aspects of the results needing attention. It is up to the individual to critically assess their own results and, if necessary, seek expert advice.

Publication of your CIF in IUCr journals

A basic structural check has been run on your CIF. These basic checks will be run on all CIFs submitted for publication in IUCr journals (*Acta Crystallographica*, *Journal of Applied Crystallography*, *Journal of Synchrotron Radiation*); however, if you intend to submit to *Acta Crystallographica Section C* or *E* or *IUCrData*, you should make sure that full publication checks are run on the final version of your CIF prior to submission.

Publication of your CIF in other journals

Please refer to the *Notes for Authors* of the relevant journal for any special instructions relating to CIF submission.

PLATON version of 13/07/2021; check.def file version of 13/07/2021

Datablock ctgu-24 - ellipsoid plot

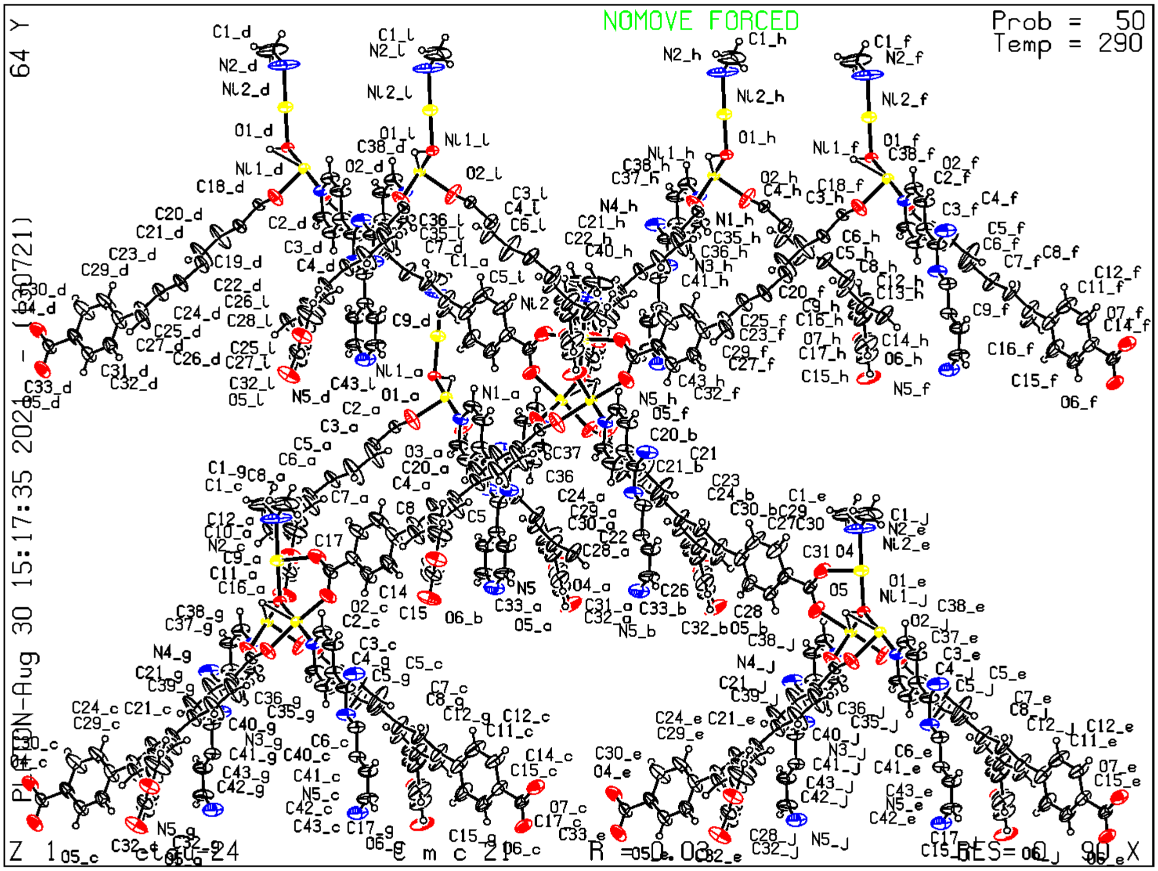

Supplement: Supplementary file 1 [file DataSheet1.ZIP › Crystallographic data/CTGU-24 checkcif.pdf]
