# Supplementary material for: In Situ Synthesis of Surface-Mounted Novel Nickel(II) Trimer-Based MOF on Nickel Oxide Hydroxide Heterostructures for Enhanced Methanol Electro-Oxidation
Source: Front Chem. 2021 Nov 29;9:780688. doi: 10.3389/fchem.2021.780688 (PMC8666528; doi:10.3389/fchem.2021.780688)
Supplement: Supplementary file 2 [file Table1.DOC]

Supplementary Material

**Novel Nickel(II) Trimer-based MOF with Mixed Tripodal Tectonics as an Active Component for Efficient Methanol Electro-oxidation**

**Ya-Ya Sun^1,‡^, Yan-Jiang Wang^1,‡^, Qiu Pi^1^, Ya-Pan Wu^1^*, Xue-Qian Wu^1^, Shuang Li^1^, Ya-Qian Lan^1^, Qichun Zhang^1^, Dong-Sheng Li^1,2^***

^1^College of Materials and Chemical Engineering, Key Laboratory of Inorganic Nonmetallic Crystalline and Energy Conversion Materials, China Three Gorges University, Yichang, Hubei, 443002, P. R. China

*^2^Hubei Three Gorges Laboratory, Yichang, Hubei 443007*

* To whom correspondence should be addressed.

E-mail: [wyapan2008@163.com](mailto:wyapan2008@163.com)

E-mail: [lidongsheng1@126.com](mailto:lidongsheng1@126.com)

# Supplementary Data

**Table S1. Crystallographic Data and Structure Refinement data for CTGU-24.**

| **Complex** | **CTGU-24** |
| --- | --- |
| Empirical formula | C_74_H_51_N_7_Ni_3_O_13_ |
| Formula weight | 1422.32 |
| Temperature/K | 293(2) |
| Crystal system | orthorhombic |
| Space group | *C*mc2_1_ |
| a/Å | 16.7054(3) |
| b/Å | 24.2139(4) |
| c/Å | 22.5829(4) |
| α/° | 90 |
| β/° | 90 |
| γ/° | 90 |
| Volume/Å^3^ | 9134.8(3) |
| Z | 4 |
| ρ_calc_g/cm^3^ | 1.035 |
| μ/mm^‑1^ | 1.144 |
| F(000) | 2932.0 |
| Crystal size/mm^3^ | 0.024 × 0.022 × 0.016 |
| Radiation | Cu K*α* (λ = 1.54184) |
| 2θ range for data collection/° | 7.302 to 148.724 |
| Index ranges | -20≤h≤19, -30≤k≤28, -27≤l≤19 |
| Reflections collected | 15249 |
| Independent reflections | 6926 [R_int_ = 0.0287, R_sigma_ = 0.0316] |
| Data/restraints/parameters | 6926/37/460 |
| Goodness-of-fit on F^2^ | 1.062 |
| Final *R* indexes [I>=2σ (I)] | *R_1_* = 0.0498, *wR_2_* = 0.1371 |
| Final *R* indexes [all data] | *R_1_* = 0.0526, *wR_2_* = 0.1397 |
| Largest diff. peak/hole / e Å^-3^ | 0.99/-1.75 |
| Flack parameter | 0.024(17) |
| CCDC No. | 2109470 |

Table S2, Selected bond lengths [Ǻ] for CTGU-24.

| **Atom** | **Atom** | **Length/Å** |  | **Atom** | **Atom** | **Length/Å** |
| --- | --- | --- | --- | --- | --- | --- |
| Ni1 | O1 | 1.988(2) |  | Ni2 | O1 | 2.032(4) |
| Ni1 | N1 | 2.116(4) |  | Ni2 | O6^1^ | 2.079(3) |
| Ni1 | O2 | 2.030(3) |  | Ni2 | O6^3^ | 2.079(3) |
| Ni1 | O3 | 2.040(3) |  | Ni2 | O4^4^ | 2.085(4) |
| Ni1 | O7^1^ | 2.027(4) |  | Ni2 | O4^2^ | 2.085(4) |
| Ni1 | O5^2^ | 2.041(4) |  | Ni2 | N2 | 2.027(6) |

Symmetry codes: ^1^1/2+*x*, 1/2-*y*, 1/2+*z*; ^2^3/2-*x*, 3/2-*y*, 1/2+*z*; ^3^1/2-*x*, 1/2-*y*, 1/2+*z*; ^4^-1/2+*x*, 3/2-*y*, 1/2+*z*; ^5^1-*x*, +*y*, +*z*; ^6^2-*x*, +*y*, +*z*.

**Table S3.** Selected bond angles [°] for CTGU-24.

| **Atom** | **Atom** | **Atom** | **Angle/˚** |  | **Atom** | **Atom** | **Atom** | **Angle/˚** |
| --- | --- | --- | --- | --- | --- | --- | --- | --- |
| O1 | Ni1 | N1 | 179.02(16) |  | O6^1^ | Ni2 | O4^2^ | 89.40(18) |
| O1 | Ni1 | O2 | 94.60(15) |  | O6^3^ | Ni2 | O4^2^ | 174.29(19) |
| O1 | Ni1 | O3 | 93.91(15) |  | O4^2^ | Ni2 | O4^4^ | 89.6(3) |
| O1 | Ni1 | O7^1^ | 92.09(15) |  | N2 | Ni2 | O1 | 176.3(2) |
| O1 | Ni1 | O5^2^ | 91.86(16) |  | N2 | Ni2 | O6^1^ | 89.47(17) |
| O2 | Ni1 | N1 | 85.33(14) |  | N2 | Ni2 | O6^3^ | 89.47(17) |
| O2 | Ni1 | O3 | 90.2(2) |  | N2 | Ni2 | O4^4^ | 84.85(17) |
| O2 | Ni1 | O5^2^ | 173.46(16) |  | N2 | Ni2 | O4^2^ | 84.85(17) |
| O3 | Ni1 | N1 | 87.07(15) |  | Ni1^5^ | O1 | Ni1 | 117.9(2) |
| O3 | Ni1 | O5^2^ | 88.4(2) |  | Ni1^5^ | O1 | Ni2 | 121.04(10) |
| O7^1^ | Ni1 | N1 | 86.94(14) |  | Ni1 | O1 | Ni2 | 121.05(10) |
| O7^1^ | Ni1 | O2 | 91.2(2) |  | C42 | N1 | Ni1 | 121.5(3) |
| O7^1^ | Ni1 | O3 | 173.71(15) |  | C40 | N1 | Ni1 | 121.2(3) |
| O7^1^ | Ni1 | O5^2^ | 89.5(2) |  | C16 | O2 | Ni1 | 133.5(3) |
| O5^2^ | Ni1 | N1 | 88.23(16) |  | C32 | O3 | Ni1 | 134.0(3) |
| O1 | Ni2 | O6^3^ | 93.14(15) |  | C1 | O7 | Ni1^6^ | 135.6(4) |
| O1 | Ni2 | O6^1^ | 93.14(15) |  | C1 | O6 | Ni2^7^ | 130.6(3) |
| O1 | Ni2 | O4^2^ | 92.52(15) |  | C17 | O5 | Ni1^8^ | 133.7(4) |
| O1 | Ni2 | O4^4^ | 92.52(15) |  | C17 | O4 | Ni2^8^ | 130.3(3) |
| O6^3^ | Ni2 | O6^1^ | 91.1(2) |  | C43^5^ | N2 | Ni2 | 122.9(4) |
| O6^1^ | Ni2 | O4^4^ | 174.29(19) |  | C43 | N2 | Ni2 | 122.9(4) |
| O6^3^ | Ni2 | O4^4^ | 89.40(18) |  |  |  |  |  |

Symmetry codes: ^1^1/2+*x*, 1/2-*y*, 1/2+*z*; ^2^3/2-*x*, 3/2-*y*, 1/2+*z*; ^3^1/2-*x*, 1/2-*y*, 1/2+*z*; ^4^-1/2+*x*, 3/2-*y*, 1/2+*z*; ^5^1-*x*, +*y*, +*z*; ^6^-1/2+*x*, 1/2-*y*, -1/2+*z*; ^7^1/2-*x*, 1/2-*y*, -1/2+*z*; ^8^3/2-*x*, 3/2-*y*, -1/2+*z*; ^9^2-*x*, +*y*, +*z*.

**Table S4.**  Comparisons of MOR performance for various electrocatalysts.

| **Electrode Materials** | **Scanning Rate**  **(mV s^-1^)** | **Peak Current Density**  **(mA cm^-2^)** | **Electrolyte** | **Reference** |
| --- | --- | --- | --- | --- |
| Ni-P/RGO | 50 | 16.4 | 1.0M KOH + 0.5M CH_3_OH | 1 |
| Mn Doped Ni(OH)_2_ | 50 | 16.7 | 1.0M KOH + 0.5M CH_3_OH | 2 |
| NiPtAu-SR_Au_ HNCs | 50 | 31.52 | 1.0M KOH + 1.0M CH_3_OH | 3 |
| NiO NTs-400 | 50 | 24.3 | 1.0M KOH + 0.5M CH_3_OH | 4 |
| PtZn intermetallic NPs | 50 | 1.15 | 0.1M KOH + 0.5M CH_3_OH | 5 |
| Pt_1_Ni_1_/C | 50 | 4.90 | 1.0M KOH + 1.0M CH_3_OH | 6 |
| NiO/Ni-P | 50 | 28.56 | 1.0M KOH + 0.5M CH_3_OH | 7 |
| NiO-SnO_2_/SO_4_^2-^ | 100 | 12.2 | 1.0M NaOH + 1.0M CH_3_OH | 8 |
| 5 wt.%GO/Co-MOF-71 | 50 | 29.1 | 1.0M KOH + 3.0M CH_3_OH | 9 |
| CTGU-24 | 50 | 14.99 | 0.1M KOH + 1.0M CH_3_OH | this work |
| CTGU-24@NiOOH | 50 | 18.75 | 0.1M KOH + 1.0M CH_3_OH |
| AB&CTGU-24@NiOOH(1:4) | 50 | 22.96 | 0.1M KOH + 1.0M CH_3_OH |
| AB&CTGU-24@NiOOH(2:4) | 50 | 29.87 | 0.1M KOH + 1.0M CH_3_OH |
| AB&CTGU-24@NiOOH(3:4) | 50 | 25.50 | 0.1M KOH + 1.0M CH_3_OH |
| AB&CTGU-24@NiOOH(4:4) | 50 | 28.27 | 0.1M KOH + 1.0M CH_3_OH |
| KB&CTGU-24@NiOOH(1:4) | 50 | 31.50 | 0.1M KOH + 1.0M CH_3_OH |
| KB&CTGU-24@NiOOH(2:4) | 50 | 27.22 | 0.1M KOH + 1.0M CH_3_OH |
| KB&CTGU-24@NiOOH(3:4) | 50 | 31.95 | 0.1M KOH + 1.0M CH_3_OH |  |
| KB&CTGU-24@NiOOH(4:4) | 50 | 34.53 | 0.1M KOH + 1.0M CH_3_OH |  |

**References**

**1**  H. Zhang, C. Gu, M. Huang, X. Wang and J. Tu, Anchoring three-dimensional network structured Ni–P nanowires on reduced graphene oxide and their enhanced electrocatalytic activity towards methanol oxidation, *Electrochem. Commun.*, 2013, **35**, 108-111.

**2** B. Dong, W. Li, X. Huang, Z. Ali, T. Zhang, Z. Yang and Y. Hou, Fabrication of hierarchical hollow Mn doped Ni(OH)_2_ nanostructures with enhanced catalytic activity towards electrochemical oxidation of methanol, *Nano Energy*, 2019, **55**, 37-41.

**3** C. Liu, Z. Chen, D. Rao, J. Zhang, Y. Liu, Y. Chen, Y. Deng and W. Hu, Behavior of gold-enhanced electrocatalytic performance of NiPtAu hollow nanocrystals for alkaline methanol oxidation, *Sci. China Mater.*, 2020.

**4** T. Wang, H. Huang, X. Wu, H. Yao, F. Li, P. Chen, P. Jin, Z. Deng and Y. Chen, Self-template synthesis of defect-rich NiO nanotubes as efficient electrocatalysts for methanol oxidation reaction, *Nanoscale*, 2019, **11**, 19783-19790.

**5**  Z. Qi, C. Xiao, C. Liu, T. W. Goh, L. Zhou, R. Maligal-Ganesh, Y. Pei, X. Li, L. A. Curtiss and W. Huang, Sub-4 nm PtZn Intermetallic Nanoparticles for Enhanced Mass and Specific Activities in Catalytic Electrooxidation Reaction, *J. Am. Chem. Soc.*, 2017, **139**, 4762-4768.

**6** S. Lu, H. Li, J. Sun and Z. Zhuang, Promoting the methanol oxidation catalytic activity by introducing surface nickel on platinum nanoparticles, *Nano Res.*, 2018, **11**, 2058-2068.

**7** Y. Tong, C. Gu, J. Zhang, H. Tang, X. Wang and J. Tu, Thermal growth of NiO on interconnected Ni–P tube network for electrochemical oxidation of methanol in alkaline medium, *Int. J. Hydrogen Energ.*, 2016, **41**, 6342-6352.

**8** Y. Gu, P. Gao, Z. Yu, Y. Hu, Z. Xu, C. Zhang, J. Li and Y. An, Honeycomb-like Mesoporous NiO-SnO_2_/SO_4_^2-^ Solid Superacid for the Efficient Reaction of Methanol Oxidation, *Int. J. Electrochem. Sci.*, 2020, **15**, 2481-2498.

**9** R. Mehek, N. Iqbal, T. Noor, H. Nasir, Y. Mehmood and S. Ahmed, Novel Co-MOF/Graphene Oxide Electrocatalyst for Methanol Oxidation, *Electrochem. Acta*, 2017, **255**, 195-204.

# Supplementary Figures and Tables

## Supplementary Figures


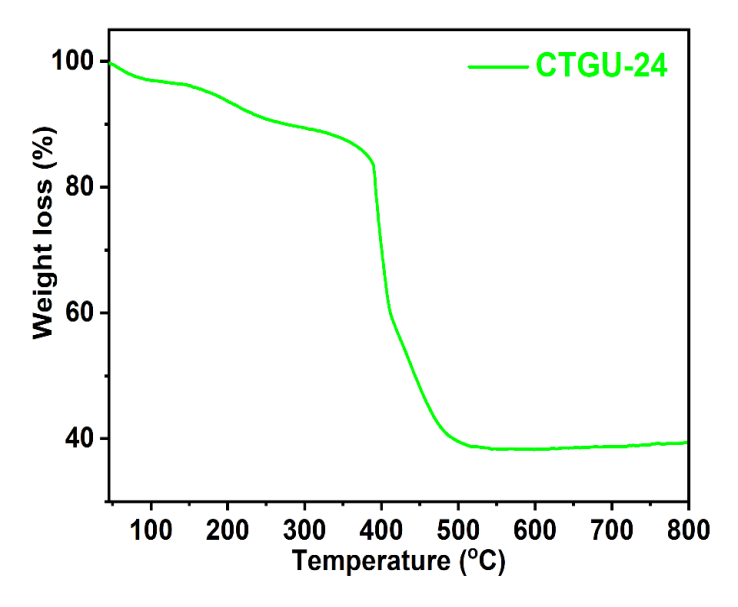


**Figure S1.** Thermogravimetric curve of CTGU-24.


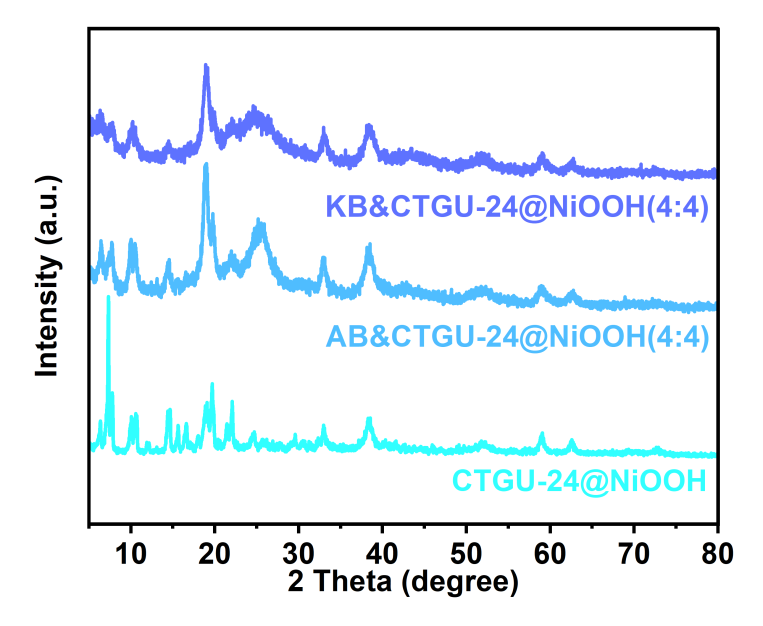


**Figure S2.** PXRD patterns of as-synthesized CTGU-24@NiOOH, AB&CTGU-24@NiOOH(4:4) and CTGU-24@NiOOH(4:4).


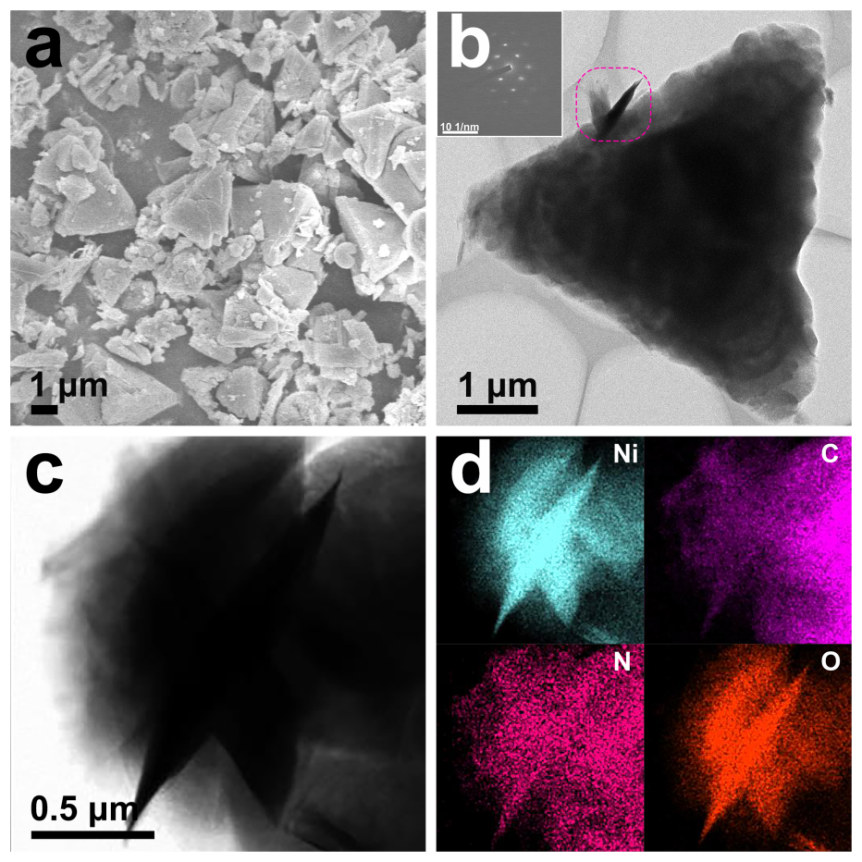


**Figure S3.** Morphological characterization of as-synthesized CTGU-24 sample. (a) SEM image. (b) TEM image, Inset in part b: Corresponding SAED pattern. (c) HRTEM image and (d) elemental mapping images of CTGU-24.

**
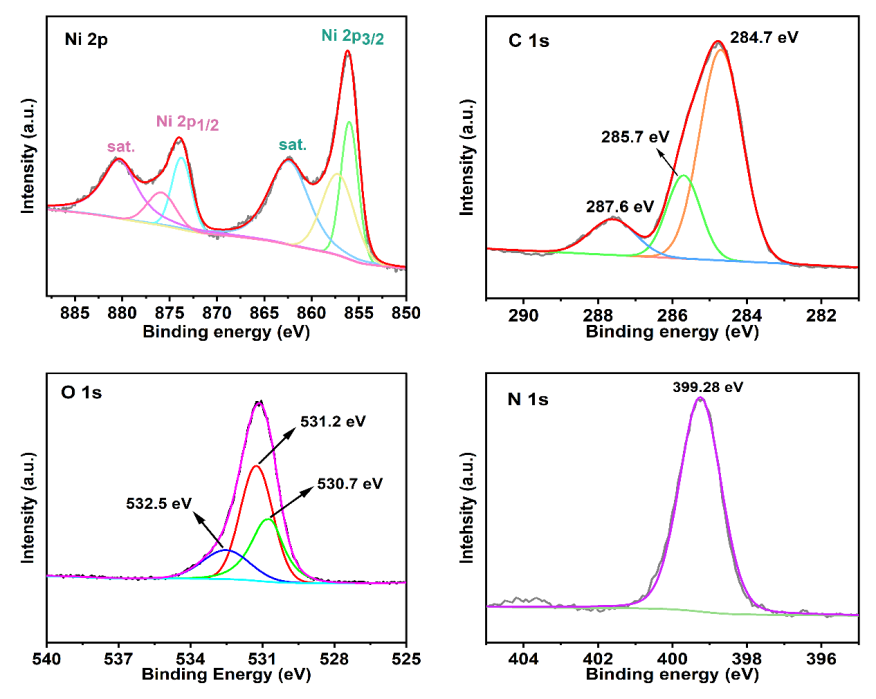
**

**Figure S4.** XPS spectra of (a) Ni 2p, (b) C 1s, (c) N 1s, and (d) O 1s regions of CTGU-24.

**
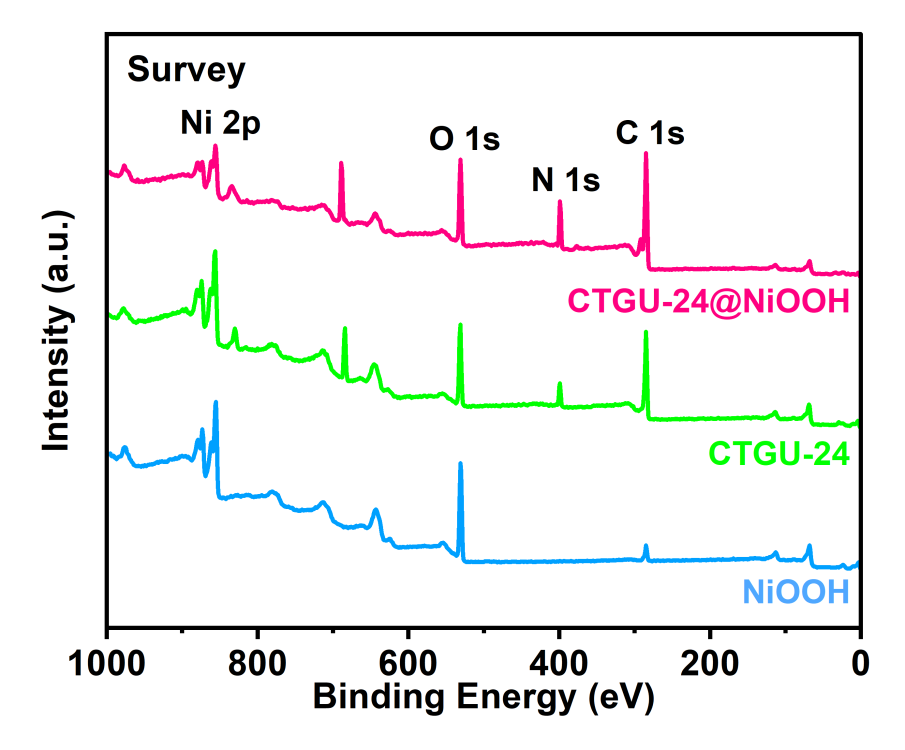
**

**Figure S5.** XPS survey spectrum of NiOOH, CTGU-24, and CTGU-24@NiOOH.

**
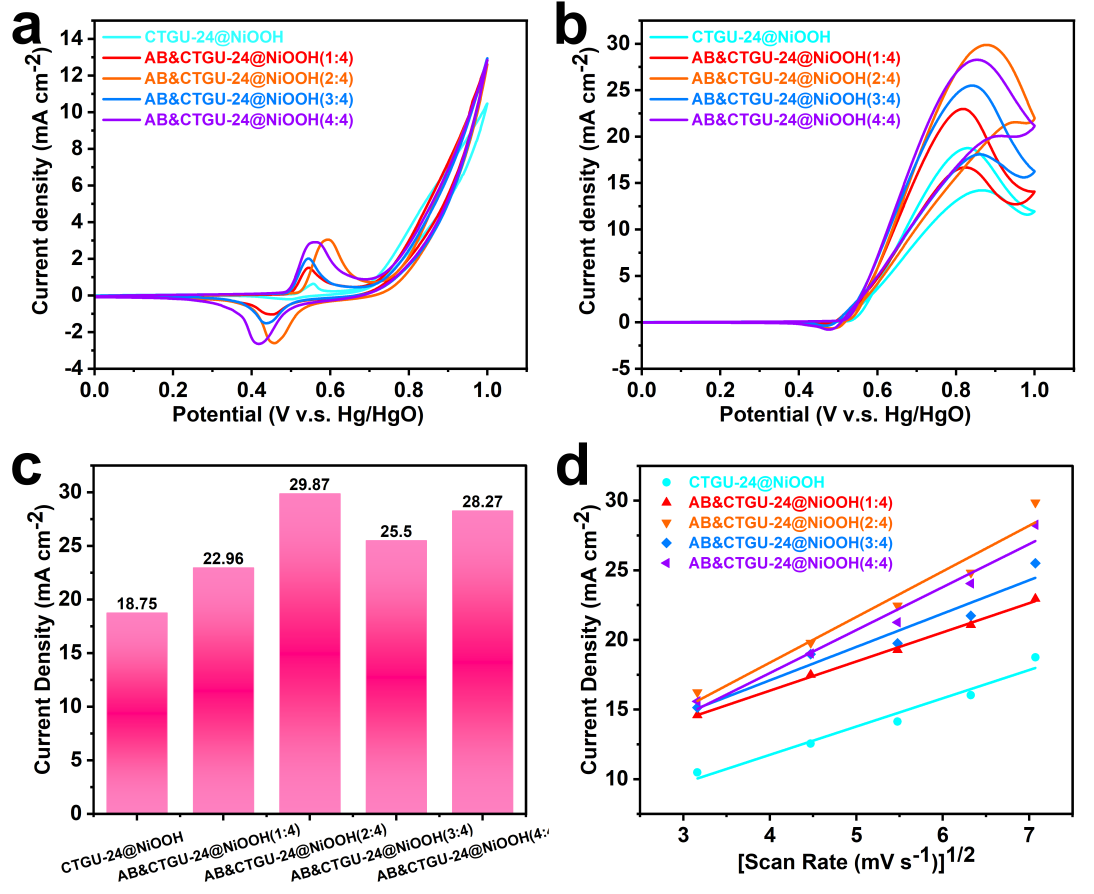
**

**Figure S6.** (a) CV curves of CTGU-24@NiOOH and AB& CTGU-24@NiOOH composite materials in 0.1 M KOH at a scan rate of 50 mV s^−1^. (b) CV curves for CH_3_OH oxidation of the CTGU-24@NiOOH and AB& CTGU-24@NiOOH composite materials in 0.1 M KOH containing 1.0 M methanol. (c) bar graph performed area activities of five catalysts. (d) view of linear correlation between current densities and square root of the scan rate.

**
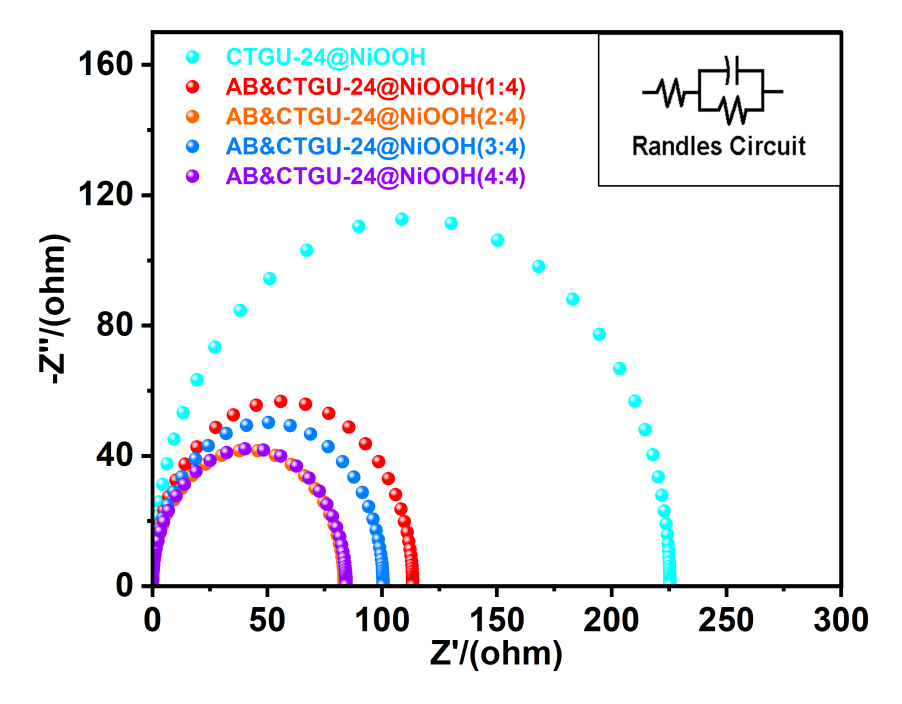
**

**Figure S7.** Nyquist curve of EIS for CTGU-24@NiOOH and AB&CTGU-24@NiOOH composite catalysts.

**
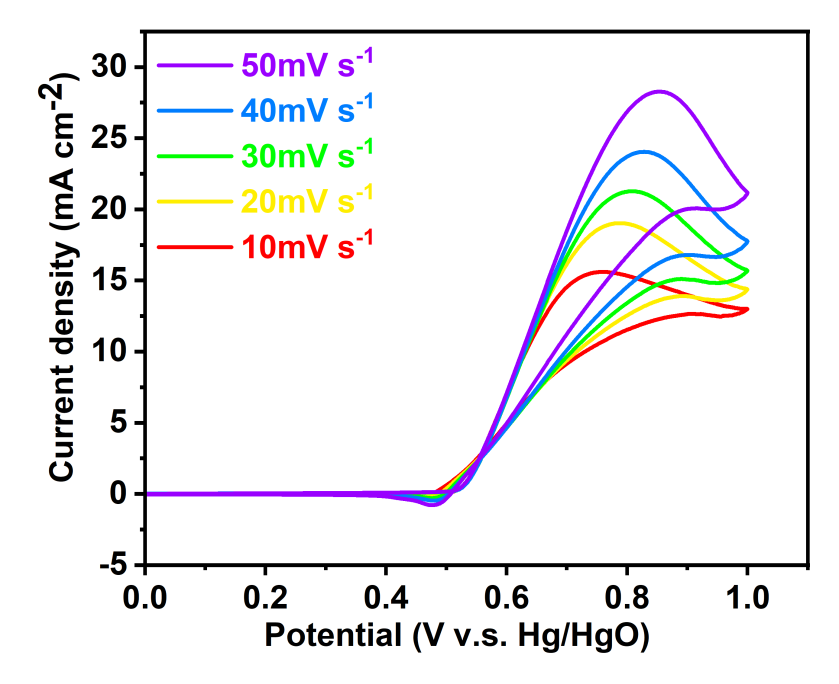
**

**Figure S8.** Cyclic voltammograms for AB&CTGU-24@NiOOH(4:4) at different scan rates.

**
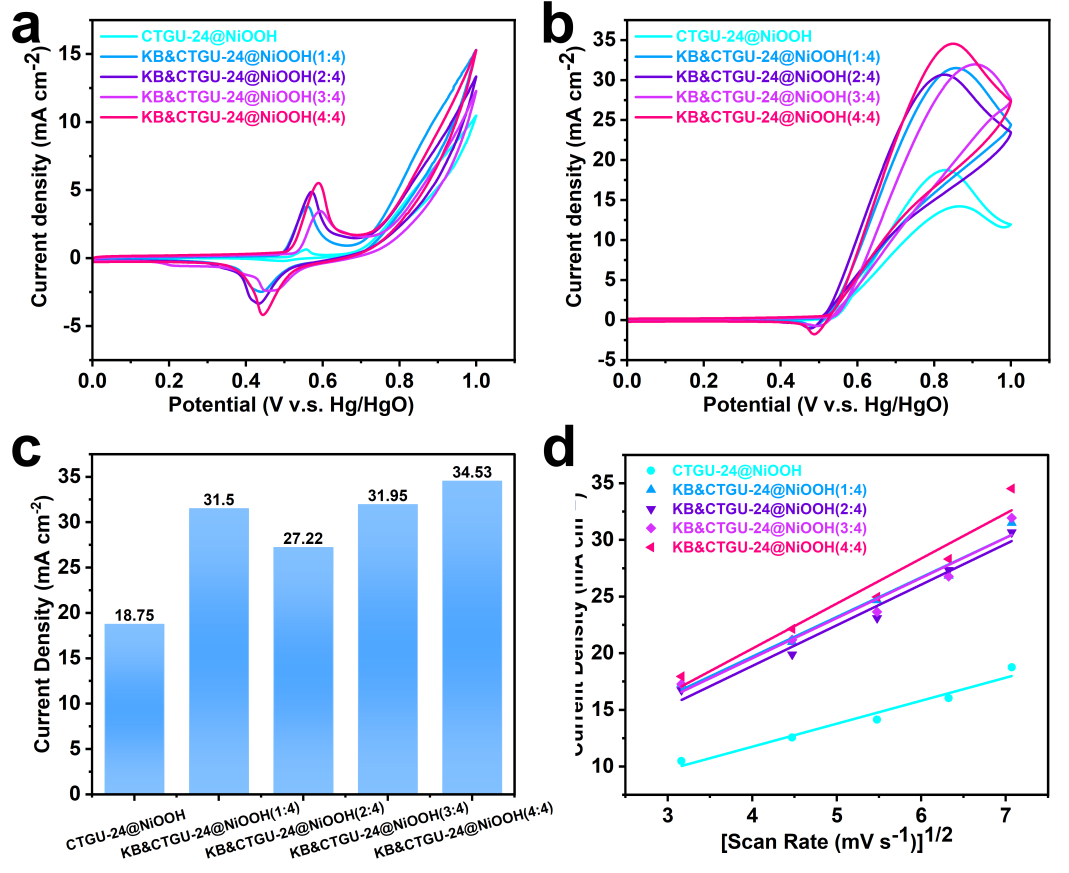
**

**Figure S9.** (a) CV curves of CTGU-24@NiOOH and KB&CTGU-24@NiOOH composite materials in 0.1 M KOH at a scan rate of 50 mV s^−1^. (b) CV curves for CH_3_OH oxidation of the CTGU-24@NiOOH and KB& CTGU-24@NiOOH composite materials in 0.1 M KOH containing 1.0 M methanol. (c) bar graph performed area activities of five catalysts. (d) view of linear correlation between current densities and square root of the scan rate.


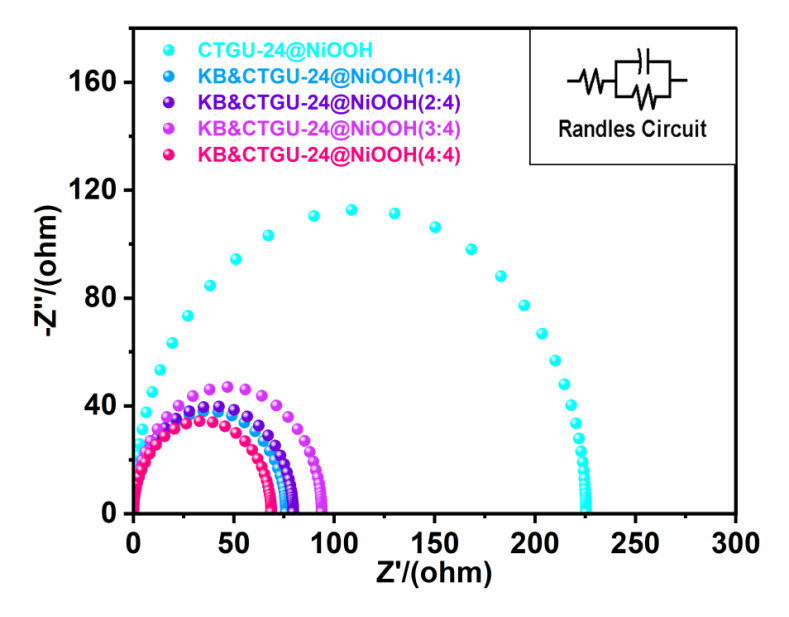


**Figure S10.** Nyquist curve of EIS for CTGU-24@NiOOH and AB&CTGU-24@NiOOH composite catalysts.


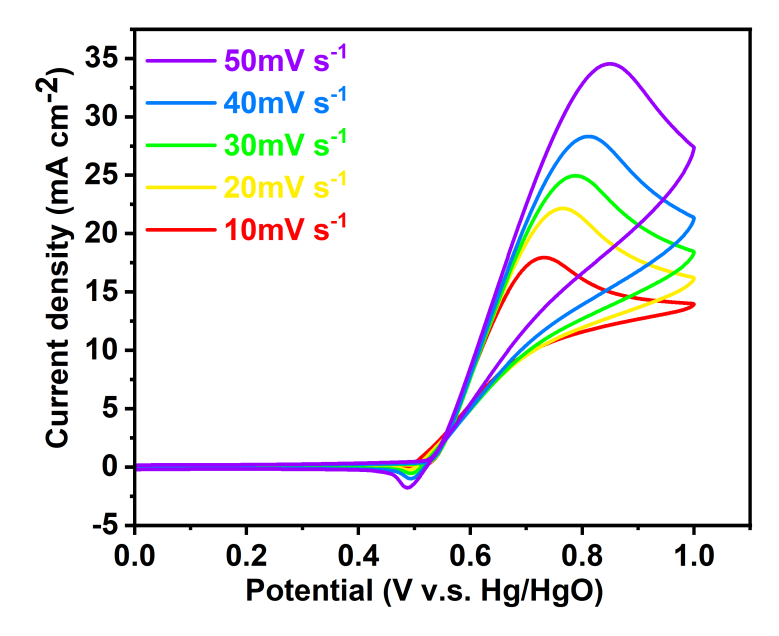


**Figure S8.** Cyclic voltammograms for KB&CTGU-24@NiOOH(4:4) at different scan rates.

**
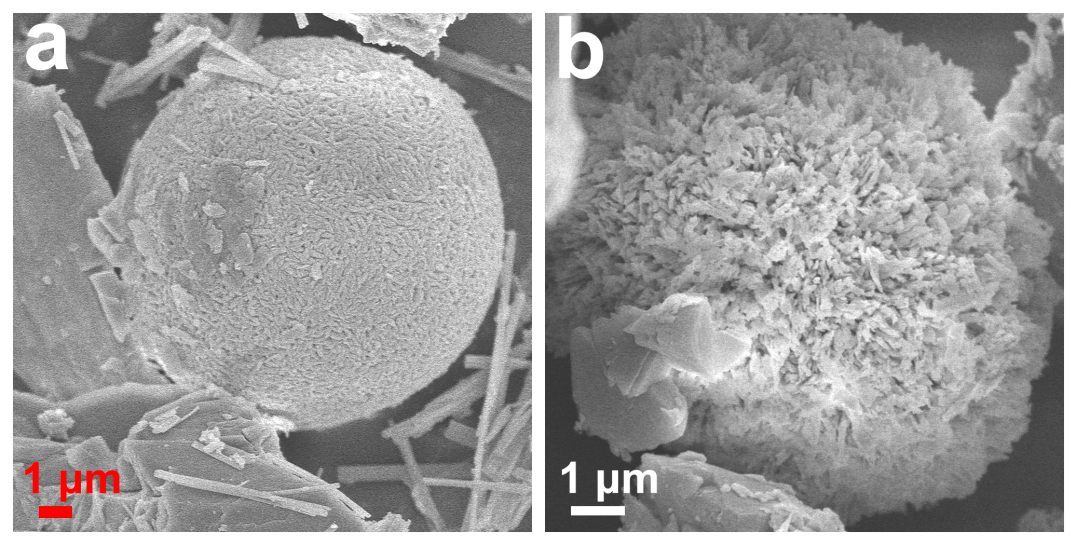
**

**Figure S9**. SEM image of KB&CTGU-24@NiOOH(4:4) hybrid material (a) before and (b) after MOR process.


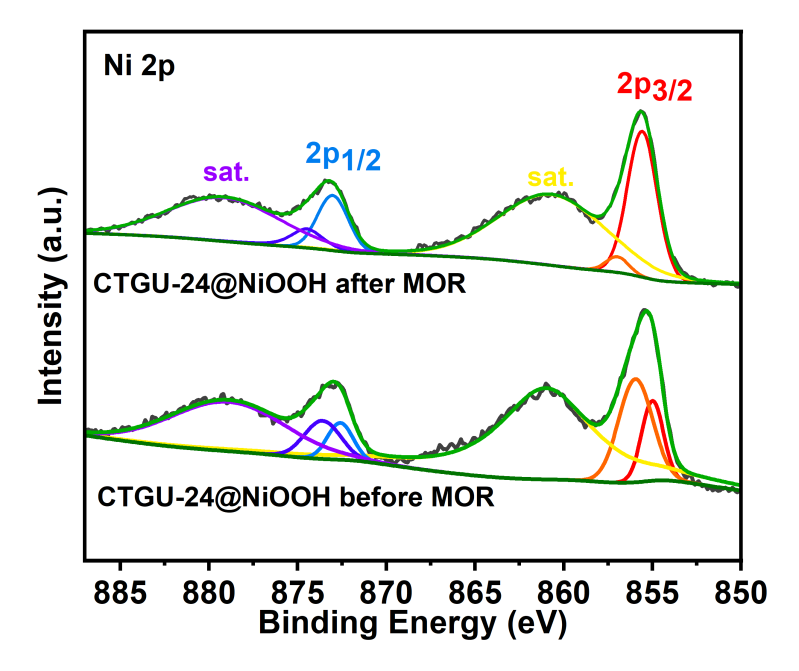


**Figure S10.** XPS Ni 2P spectra of the CTGU-24@NiOOH catalyst before and after MOR.

**
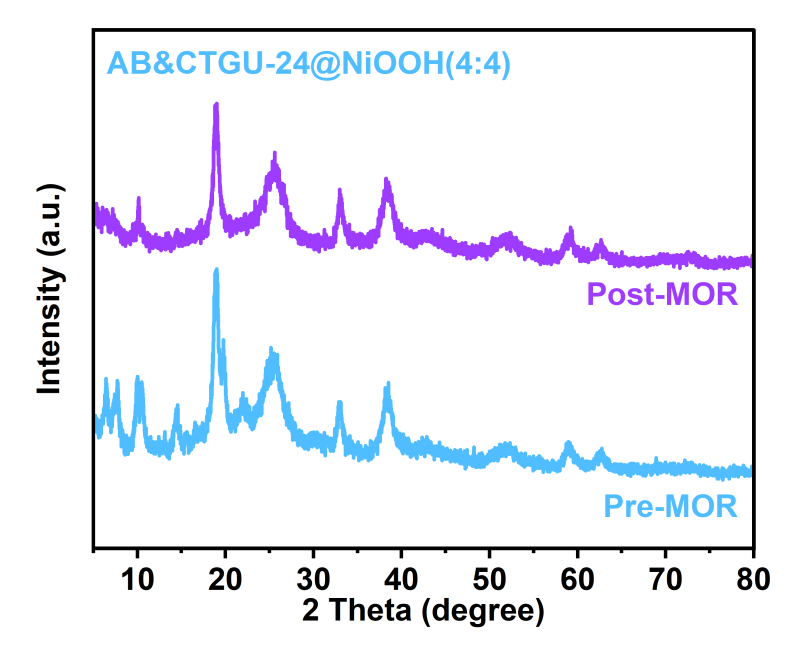
**

**Figure S11.** PXRD curves of as-synthesized AB&CTGU-24@NiOOH pre- and post-MOR test.

**
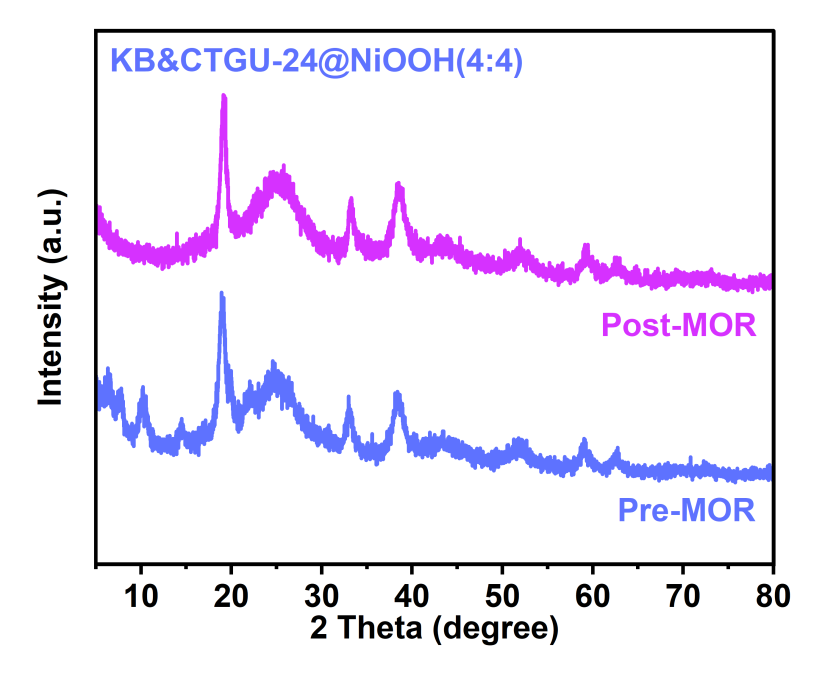
**

**Figure S12.** PXRD curves of as-synthesized KB&CTGU-24@NiOOH(4:4) pre- and post-MOR test.
